# Thoracoscopic segmentectomy for lung tumour with displaced B^1 + 2^

**DOI:** 10.1093/jscr/rjae810

**Published:** 2024-12-26

**Authors:** Masahiro Miyajima, Keishi Ogura, Taijirou Mishina, Atsushi Watanabe

**Affiliations:** Department of Thoracic Surgery, Sapporo Medical University, Sapporo, Japan; Division of Radiology and Nuclear Medicine, Sapporo Medical University, Sapporo, Japan; Department of Thoracic Surgery, Sapporo Medical University, Sapporo, Japan; Department of Thoracic Surgery, Sapporo Medical University, Sapporo, Japan

**Keywords:** video-assisted thoracic surgery, displaced bronchus, three-dimensional contrast-enhanced computed tomography

## Abstract

The frequency of bronchial branching abnormalities is about 0.6%, of which about 75% are related to the right upper lobe. The frequency of left B^1 + 2^ transition bronchus is even rarer, but a few cases have been reported. A 43-year-old man, who presented with an abnormal pulmonary nodule suspected to be lung cancer in the left S4 segment, underwent video-assisted thoracoscopic segmentectomy of S3 plus lingular segment. Preoperative three-dimensional contrast-enhanced computed tomography (CT) revealed a displaced B^1 + 2^ bronchus arising from the left main bronchus, which ascends behind the main pulmonary artery. Video-assisted thoracic surgery was performed successfully, and the nodule was pathologically diagnosed as a lymphoma. Preoperative three-dimensional contrast-enhanced CT was very useful to detect this rare bronchial abnormality. In the present case, three-dimensional CT allowed us to safely operate on a patient with a rare B^1 + 2^ displaced bronchus in the left upper lobe.

## Introduction

The frequency of bronchial branching abnormalities is about 0.6%, of which about 75% are related to the right upper lobe. The frequency of left B^1 + 2^ transition bronchus is even rarer, but a few cases have been reported.

## Case

**Figure 1:**
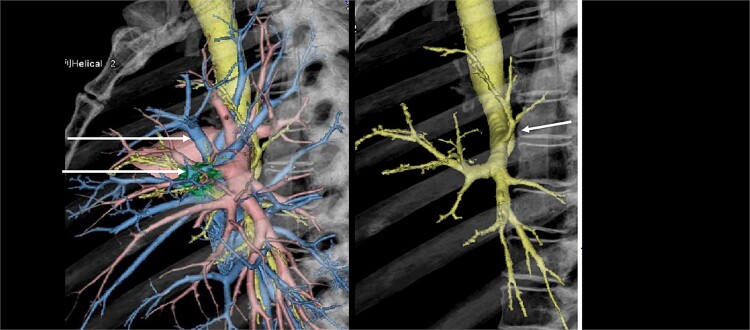
Preoperative three-dimensional contrast-enhanced computed tomographies (3DCT) showed a displaced anomalous B1^+2^ bronchus arising from left main bronchus, which ascends behind the main pulmonary artery.

**Figure 2:**
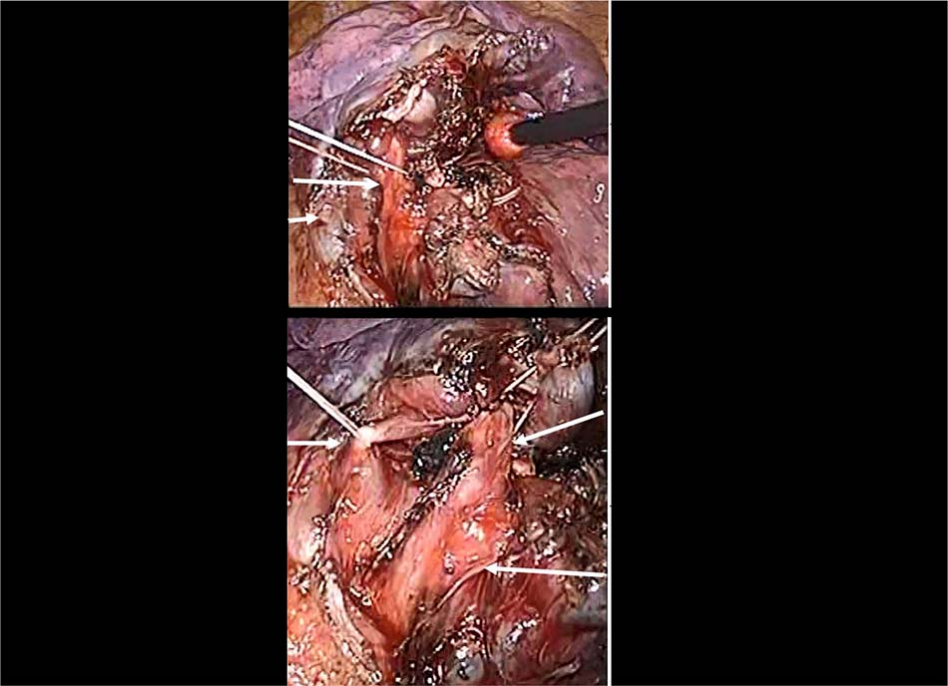
Intraoperative image showing the B3 plus lingular bronchus. Owing to the understanding of the relative anatomy of arteries, veins, and bronchi, posterior fissure making was not necessary. Br, bronchus; PV, pulmonary vein.

A 43-year-old man, who presented with an abnormal pulmonary nodule suspected to be lung cancer in the left S4 segment, underwent video-assisted thoracoscopic segmentectomy of S3 plus lingular segment. Preoperative three-dimensional contrast-enhanced computed tomography (CT; Fig. 1) revealed a displaced B^1 + 2^ bronchus arising from left main bronchus, which ascends behind the main pulmonary artery. Video-assisted thoracic surgery was performed successfully, and the nodule was pathologically diagnosed as a lymphoma (Fig. 2). Preoperative three-dimensional contrast-enhanced CT is very useful to detect this bronchial abnormality. The post-operative course was good, and the thoracic drain was removed on the first post-operative day. The patient was discharged on the sixth post-operative day.

## Discussion

Lung cancer is one of the most common cancers worldwide in terms of incidence and mortality. It is also one of the most difficult cancers to manage, and treatment development is actively being pursued. Recently, with the spread of cancer screening and novel CT techniques, more opportunities for early detection of lung cancer have become available, and surgery is becoming increasingly important as a curative treatment. As life expectancy and the incidence of new cancers increase, the importance of preserving lung function has been recognised. Therefore, the extent of lung resection in early-stage lung cancers continues to be debated.

In recent years, as the diagnostic accuracy for lung cancer has improved, the proportion of small lesions in surgical cases has increased. Based on the results of the JCOG0802 and CALGB140503 trials, which demonstrated the superiority of sublobar resections for early nonsmall cell lung cancer, the need for sublobar resections is expected to increase in the future [1, 2]. Many studies have reported the effectiveness of sublobar resection and the usefulness of preoperative CT in determining the margins for sublobar resection [3, 4].

In this situation, bronchial abnormalities have a significant impact on the safety of pulmonary sementectomy. Foster–Carter classified bronchial abnormalities into three categories: supernumerary bronchus, in which excessive bronchi branching occurs in addition to normal bronchial branching; displaced bronchus, in which bronchi branch away from their original position; and congenital cystic disease (congenital cystic disease) [5]. In this case, B^1 + 2^ was classified into the displaced bronchus. The segmental plane was divided along with the pulmonary veins, but the use of indocyanine green was also considered feasible. We did not need to complete posterior fissure, which reduced the operative time and the risk of post-operative air leak [6]. Although there are few reports of lung resection for patients with abnormal bronchus, the pre-operative 3DCT study of the anatomy allowed us to safely perform the surgery as simulated.

## Conclusions

In the present case, three-dimensional CT allowed us to safely operate on a patient with a rare B^1 + 2^ displaced bronchus in the left upper lobe (Fig. 1). To the best of our knowledge, this is the first report of such an abnormality. We believe that this report contributes to the safety of an increasing number of sublobar resections.
